# Monolithic Multi Degree of Freedom (MDoF) Capacitive MEMS Accelerometers

**DOI:** 10.3390/mi9110602

**Published:** 2018-11-16

**Authors:** Zakriya Mohammed, Ibrahim (Abe) M. Elfadel, Mahmoud Rasras

**Affiliations:** 1 Department of Electrical and Computer Engineering, New York University-Tandon School of Engineering, Brooklyn, NY 11201, USA; 2 Department of Electrical and Computer Engineering, Khalifa University, Abu Dhabi 54224, UAE; ibrahim.elfadel@ku.ac.ae; 3 Engineering Department, New York University, Abu Dhabi 129118, UAE; mrasras@nyu.edu

**Keywords:** accelerometer, multi-axis sensing, capacitive transduction, inertial sensors, three-axis accelerometer, micromachining, miniaturization

## Abstract

With the continuous advancements in microelectromechanical systems (MEMS) fabrication technology, inertial sensors like accelerometers and gyroscopes can be designed and manufactured with smaller footprint and lower power consumption. In the literature, there are several reported accelerometer designs based on MEMS technology and utilizing various transductions like capacitive, piezoelectric, optical, thermal, among several others. In particular, capacitive accelerometers are the most popular and highly researched due to several advantages like high sensitivity, low noise, low temperature sensitivity, linearity, and small footprint. Accelerometers can be designed to sense acceleration in all the three directions (X, Y, and Z-axis). Single-axis accelerometers are the most common and are often integrated orthogonally and combined as multiple-degree-of-freedom (MDoF) packages for sensing acceleration in the three directions. This type of MDoF increases the overall device footprint and cost. It also causes calibration errors and may require expensive compensations. Another type of MDoF accelerometers is based on monolithic integration and is proving to be effective in solving the footprint and calibration problems. There are mainly two classes of such monolithic MDoF accelerometers, depending on the number of proof masses used. The first class uses multiple proof masses with the main advantage being zero calibration issues. The second class uses a single proof mass, which results in compact device with a reduced noise floor. The latter class, however, suffers from high cross-axis sensitivity. It also requires very innovative layout designs, owing to the complicated mechanical structures and electrical contact placement. The performance complications due to nonlinearity, post fabrication process, and readout electronics affects both classes of accelerometers. In order to effectively compare them, we have used metrics such as sensitivity per unit area and noise-area product. This paper is devoted to an in-depth review of monolithic multi-axis capacitive MEMS accelerometers, including a detailed analysis of recent advancements aimed at solving their problems such as size, noise floor, cross-axis sensitivity, and process aware modeling.

## 1. Introduction

An accelerometer is a mechanical sensor which measures various modes of accelerations whether they are constant (gravity), time varying (vibrations), or quasi static (tilt). The miniaturization of these sensors was triggered with the advent of microelectromechanical systems (MEMS) technology in the late 1960s and early 1970s [1]. MEMS have had a great positive impact on growing the applications of accelerometers to domains ranging from automotive to biomedical [2,3]. Apart from accelerometers, many sensors have used MEMS technology for miniaturization, including pressure sensors, gyroscopes, micromirrors, and microphones [4].

To accurately determine the motion and position of an object in space, a microsystem must have 10 degrees of freedom (DOF) [5]. This condition can be fulfilled by using a combined system of three-axis accelerometer (3 DOF), three-axis gyroscope (3 DOF), three-axis magnetometer (3 DOF), and a barometer (1 DOF). Therefore, for a high precision inertial navigation system, an accelerometer with three-axis sensing is desired. Most of the reported accelerometers are either single-axis or two-axis and for sensing motion in three directions, assembling two or three of these accelerometers is typically undertaken. The simplest assembly approach is orthogonal mounting and packaging of three single-axis accelerometers. However, there are many drawbacks in such assembly, including larger device footprint, higher packaging cost, and increased chances of misalignment errors [6,7]. These misalignment errors require corrections and compensations which further increases the cost.

Monolithic three-axis accelerometers seem to solve many of the issues related to expensive packaging, misalignment errors, and size. There are several approaches for the monolithic implementation of a three-axis accelerometer, including

1. Single chip integration of three proof masses, each sensing a particular axis.
2. Monolithic fabrication of two proof masses, one for in-plane sensing (X and Y) and the other for out-of-plane sensing (Z-axis).
3. Single proof mass designed to sense all the three directions.

Monolithic three-axis accelerometers that are composed of multiple proof masses have been reported since 1990s [8]. These devices have very low cross-axis sensitivity but suffer from high Brownian noise and have relatively large form factor [8]. On the other hand, it has been found that with the use of a single proof mass for three-axis sensing, a 50% reduction in the chip size can be achieved [9,10]. Even though their Brownian noise is low, the single proof-mass accelerometers suffer from very high cross-axis sensitivity. Moreover, complex innovative designs are needed for sensing all the three directions with only a single proof mass. The main objective of the present paper is to survey the reported monolithic multi-axis accelerometers and analyze in detail their structures and key MEMS design decisions that have enabled them to overcome the reported sensing challenges.

## 2. Applications of Multi-Axis Accelerometers

Miniaturized multi-axis accelerometers are mainly used in inertial measurement units (IMUs), along with gyroscopes and magnetometers for position and motion sensing. However, there use is increasing in many consumer electronic applications. Accelerometers are incorporated in electronics such as digital cameras, smart phones, notebooks, and video games. Apple iPhone 3G [11], Google Nexus One [12], Nokia N97 [13], and Nintendo Wii [14], all had three-axis accelerometers. In the current generation of these smart devices, the board size to mount the sensors is decreasing while the number of mounted sensors is increasing. Therefore, it is necessary to constantly pursue research for reducing the accelerometer footprint while enhancing its performance. In these smart gadgets, the accelerometer performs various functions, including flipping the display according to changes in gadget orientations, image stabilization while taking photos, better user experience while playing video games, and detecting whether the gadget is at rest of under free fall. This feature of detecting free fall is used to protect data in a notebook by quickly turning off the hard drive during accidental drops.

In industry, three-axis accelerometers are used for motion control, robot positioning, finding incline angles of bulky structure, and vibration monitoring. In healthcare, they are used to monitor fibrillation and arrhythmias during heart surgery. Heavy vehicles such as pickup trucks and sports utility vehicles have a very high center of gravity making them more susceptible to rollover accidents. Therefore, a three-axis accelerometer is used to detect rollovers and deploy side airbags. Some of the advanced applications of multi-axis accelerometers include electronic stability control, automotive headlight leveling and vehicle alarm. 

## 3. Accelerometer Operating Principle

**Figure 1:**
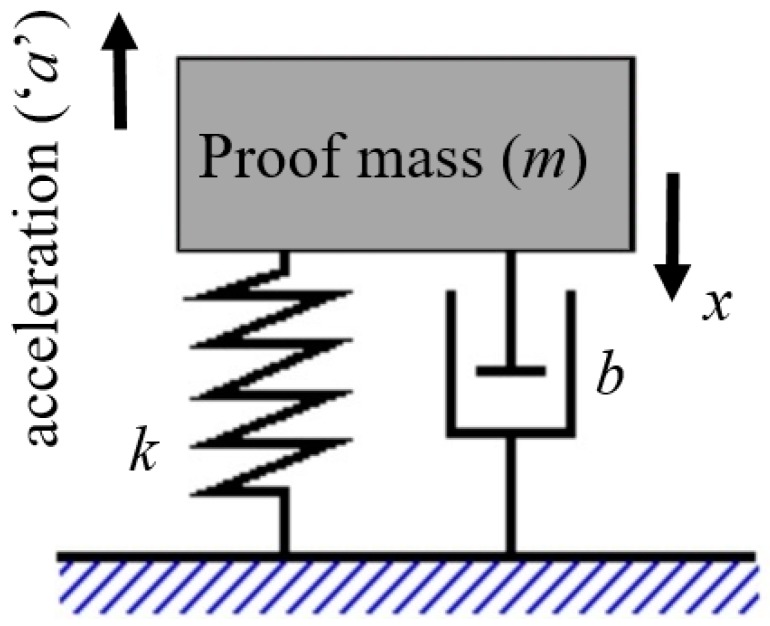
Model of accelerometer.

An accelerometer can be modeled as a second order spring-mass-damper system (Figure 1). When an acceleration (*a*) is applied to proof mass (*m*) suspended by springs with a spring constant (*k*), and having a damping (*b*), then the force (*F_applied_*) acting on the proof mass is given by:

(1) $$F_{applied}=ma_{applied}$$

The force exerted by springs and damping in the system can be defined as:

(2) $$F_{spring}=kx$$

(3) $$F_{damping}=b\dot{x}$$

Applying Newton’s second law which states that the algebraic sum of all the forces equals the inertial force of the proof mass, we get:

(4) $$F_{applied}-F_{spring}-F_{damping}=m\ddot{x}$$

(5) $$m\ddot{x}+b\dot{x}+kx=F_{applied}=ma_{applied}$$

The transfer function *H*(*s*) of the system is given by:

(6) $$ms^{2}x\left(s\right)+bsx\left(s\right)+kx\left(s\right)=F\left(s\right)=ma\left(s\right)$$

(7) $$s^{2}x\left(s\right)+\frac{b}{m}sx\left(s\right)+\frac{k}{m}x\left(s\right)=\frac{F\left(s\right)}{m}=a\left(s\right)$$

(8) $$H\left(s\right)=\frac{x\left(s\right)}{a\left(s\right)}=\frac{1}{s^{2}+\frac{b}{m}s+\frac{k}{m}}=\frac{1}{s^{2}+\frac{\omega_{0}}{Q}s+\omega_{0}{}^{2}}$$

In Equation (8), $\omega_{0}$ is the resonance frequency and *Q* is the quality factor given by:

(9) $$\omega_{0}=\sqrt{k/m}$$

(10) $$Q=\frac{m\omega_{0}}{b}$$

Accelerometers work in the low frequency domain ($\omega \ll \omega_{0})$ with their mechanical sensitivity calculated by setting *s* = 0 in the transfer function *H*(*s*) to get

(11) $$\frac{x}{a}\;\sim \;\frac{m}{k}=\frac{1}{\omega_{0}{}^{2}}$$

In order to have a large sensing bandwidth, we need a high resonant frequency which can be achieved by reducing the size of the proof mass and increasing the stiffness of the springs. However, this reduces the sensitivity of the device. Therefore, there is a tradeoff between the sensitivity and bandwidth.

## 4. Specifications of Accelerometers

MEMS accelerometers are used for various kinds of applications and therefore their specifications are application dependent. For example, in seismic measurements, accelerometers with an operation range greater than ±0.1 g, frequency range of 0–1 Hz, and resolution less than 1 μg are required. On the other hand, in shock or impact sensing, they require a range of 10,000 g, a resolution less than 1 g, and a bandwidth of 50 kHz. In this section, we give a brief overview of the specifications of an accelerometer and the design parameters on which they depend. Accelerometers are typically characterized by their Brownian noise, sensitivity, frequency response, resolution, nonlinearity, range, cross-axis sensitivity, and shock resistance. 

### 4.1. Brownian Noise

One of the most important factor to be considered during the design is the Brownian noise. It limits the minimum achievable resolution of an accelerometer. Brownian noise is given by:

(12) $$\sqrt{\frac{a_{n}^{2}}{\Delta f}}=\frac{\sqrt{4K_{B}Tb}}{m}=\sqrt{\frac{4K_{B}T\omega }{mQ}}$$

where

*a_n_* = Brownian equivalent acceleration noise
Δf = Bandwidth
$K_{B}$ = Boltzmann constant
*T* = Absolute temperature in Kelvin

From Equation (12), it is clear that lower noise can be achieved with larger proof mass and higher quality factor. In a single proof-mass three-axis accelerometer, a relatively large proof mass is used to sense acceleration in all the three directions. Therefore, it will have lesser noise compared to three-axis accelerometer formed by the integration of three smaller one-axis accelerometers. The noise floor in the later can also be reduced by increasing the individual size of each proof mass but this will drastically increase the overall footprint.

### 4.2. Sensitivity

The sensitivity of an accelerometer is defined as the output voltage signal generated per unit input acceleration in ‘g’. It is sometimes referred to as scale factor and denoted by ‘*S*’. The general units are mV/g. For a triaxial accelerometer, the axial sensitivities are independent along the X, Y and Z axes are denoted by *X_S_*, *Y_S_* and *Z_S_*.

(13) $$X_{S}=\frac{Output\;Volage\;generated\;\left(\mathrm{mV}\right)}{input\;acceleration\;along\;X-axis\;\left(g\right)}$$

(14) $$Y_{S}=\frac{Output\;Volage\;generated\;\left(\mathrm{mV}\right)}{input\;acceleration\;along\;Y-axis\;\left(g\right)}$$

(15) $$Z_{S}=\frac{Output\;Volage\;generated\;\left(\mathrm{mV}\right)}{input\;acceleration\;along\;Z-axis\;\left(g\right)}$$

### 4.3. Cross-Axis Sensitivity

Cross-axis sensitivity is the output voltage generated due to an acceleration orthogonal to a sensitive axis. Cross-axis sensitivity is generally expressed in percentage of the sensitivity i.e., ratio of the measured voltage in the cross-axis direction to the measured voltage in the sensing axis. For a tri-axial accelerometer, each axis has two cross-axis sensitivities. For example, in the case of X-direction sensing axis, there is cross-axis sensitivity due to Y-axis acceleration (*X_s_*)*_AY_* and Z-axis acceleration (*X_s_*)*_AZ_*.

(16) $${\left(X_{S}\right)}_{AY}=\frac{Output\;Volage\;generated\;\left(\mathrm{mV}\right)}{input\;acceleration\;along\;Y-axis\;\left(g\right)}$$

(17) $${\left(X_{S}\right)}_{AZ}=\frac{Output\;Volage\;generated\;\left(\mathrm{mV}\right)}{input\;acceleration\;along\;Z-axis\;\left(g\right)}$$

A three-axis single proof-mass accelerometer can move freely in the three directions and the proof-mass displacement is directly proportional to the output voltage. It is therefore prone to high cross-axis sensitivity. On the other hand, a single-axis accelerometer has high stiffness in the cross direction and thus has very low cross-axis sensitivity. Therefore, the monolithic integration of multiple proof masses has a similar advantage.

### 4.4. Dynamic Range and Nonlinearity

The dynamic range of the accelerometer is the maximum dynamic acceleration that can be measured accurately. It is given in ‘±g’.

The output response of an ideal accelerometer is linear with the input acceleration. The nonlinearity of the accelerometer, therefore, measures the deviation in the output signal with respect to the ideal linear sensitivity behavior. It is expressed in terms of full-scale range as

(18) $$\%\;Non\;linearity=\frac{Maximum\;deviation\;\left(g\right)}{Full\;scale\;range\;\left(g\right)}\times 100$$

### 4.5. Frequency Response and the Bandwidth

The frequency response gives the dependence of accelerometer sensitivity on frequency. It also gives the amplitude and phase responses of the accelerometer. The sensitivity of an accelerometer remains constant below the resonant frequency. The range of frequencies in which the sensitivity remains constant within a tolerance band of ±3 dB is the 3 dB bandwidth of the accelerometer

## 5. Types of Accelerometers

**Figure 2:**
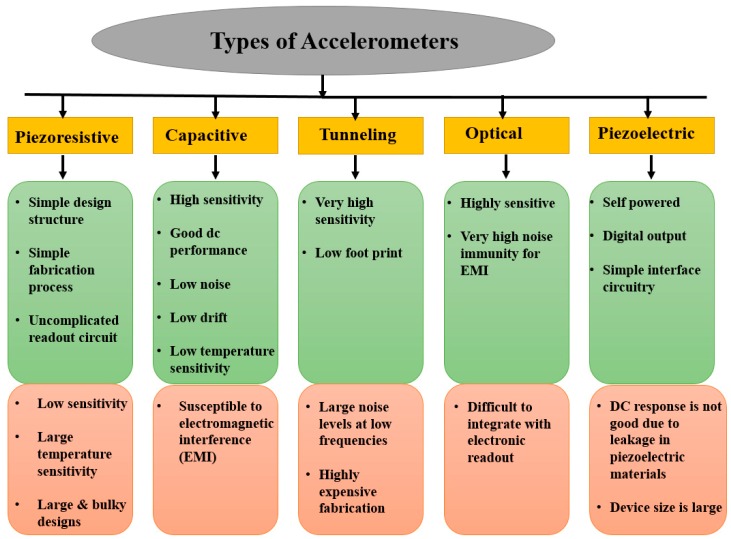
Advantages and disadvantages of various transduction schemes.

Depending on the transduction mechanism employed to convert the proof-mass displacement due to acceleration into a measurable signal, accelerometers can be classified as Piezoresistive [15], Piezoelectric [16], capacitive, resonant [17], optical [18], thermal [19], and tunneling [20]. The advantages and disadvantages of these transductions are explained in Figure 2.

### 5.1. Capacitive Accelerometers

In capacitive accelerometers, the displacement in the proof mass due to acceleration is converted to a proportional capacitance change, which is later converted and amplified into a voltage signal. There are rotor electrode plates attached to the proof mass and stator electrode plates attached to the substrate. The design of a capacitive accelerometer is accomplished so as to have a simultaneous capacitance increase and decrease with the same acceleration with differential sensing traditionally used for quantifying the acceleration. Differential sensing increases the sensitivity by a factor of 2.

In order to fabricate capacitive accelerometers, there are two basic processes: surface and bulk micromachining. In surface micromachining, the accelerometer structure is fabricated on top of the substrate [21,22]. This is done by using various film deposition techniques similar to that of complementary metal-oxide-semiconductor (CMOS) fabrication. Therefore, the main advantage of this method lies in excellent CMOS compatibility. In this process, the first step is to deposit and pattern a sacrificial layer on the substrate, followed by the deposition and patterning of a structural layer on top. The sacrificial layer is subsequently etched, releasing a suspended mechanical structure. The devices fabricated using surface-micromachining suffers from high noise due to the thin structural layer thickness, and high internal stresses.

In contrast, bulk-micromachining uses etching of the bulk silicon substrate to create a suspended structure within the wafer [23]. The etching can be done using either wet (isotropic/anisotropic) or dry etching techniques. In isotropic etching, the etch rate is the same in all directions while in anisotropic etching the rate differs according to crystal orientation. For high aspect ratio structures, reactive ion etching (RIE) or deep reactive ion etching (DRIE) techniques are used. The structures realized using the bulk micromachining process have the advantages of low noise due to thick structures and good stability but have the drawbacks of higher cost and complex fabrication.

Apart from these two basic fabrication processes, there are new fabrication techniques that are meant to overcome the various drawbacks. Some of them utilize advantages of both surface and bulk micromachining while others use nonstandard methods for surface micromachining such as electroplating through resist molds [24]. Electroplating mitigates the disadvantage of surface micromachining by increasing the thickness of the structure. There are also reported developments for monolithically fabricating a three-axis accelerometer, a three-axis gyroscope, and a three-axis magnetometer in a single chip [25,26].

#### 5.1.1. In-Plane Capacitive Accelerometers

**Figure 3:**
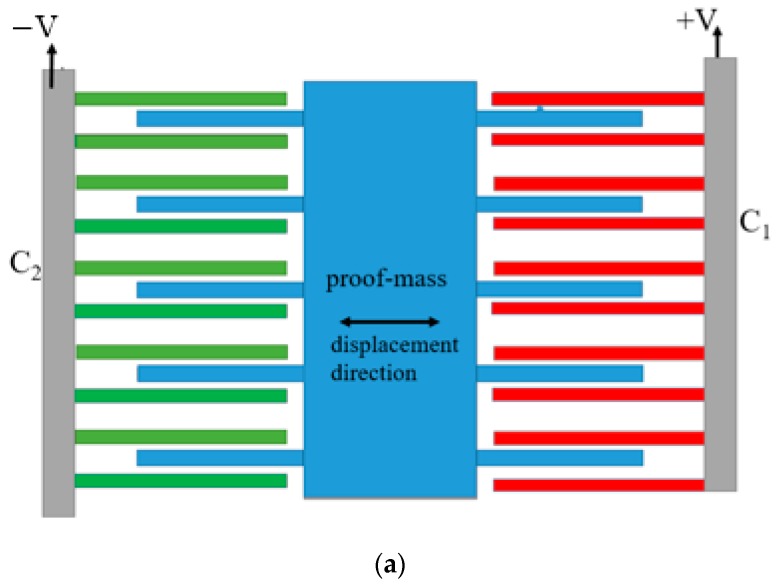

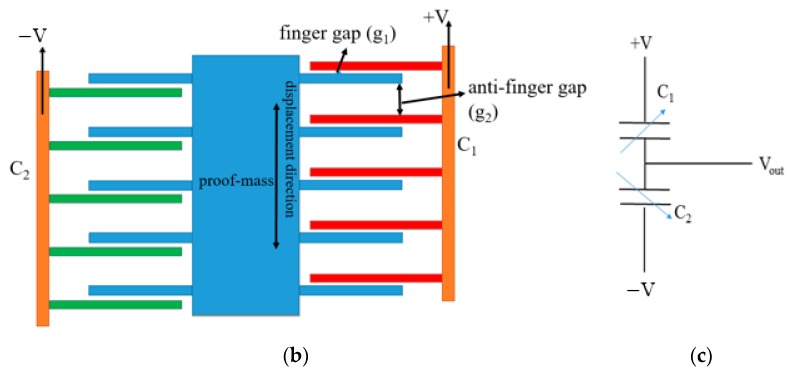
Sensing scheme of (**a**) area change accelerometer (**b**) gap change accelerometer (**c**) equivalent circuit.

The in-plane acceleration (X-axis, Y-axis or both) is generally sensed using multiple comb electrodes attached to the proof mass (rotors) and combs fixed to the substrate (stators). Since the capacitance is directly proportional to overlap area between the combs and inversely proportional to finger gap, the accelerometer can be designed to generate capacitance change with acceleration in either of two ways i.e., change in the overlap area between combs (Area Change) [27] or change in the gap between rotor and stator combs (Gap Closing). In the first approach, the capacitance changes linearly with the displacement. However, it results in a very small fractional change in capacitance. This approach is not used very often because of its low sensitivity. Figure 3a shows a block diagram of the area changeable accelerometer. With the application of an external acceleration, the proof mass is displaced, causing capacitance change in the right and left-hand side combs. On one side, the capacitance ‘C_1_’ increases while on the other, capacitance ‘C_2_’ decreases under the same acceleration. The stator fingers are excited with a differential voltage (opposite polarity) to produce an output signal which is proportional to the capacitance difference (C_1_–C_2_).

The second approach, which is based on the change in the gap spacing between the two plates [28,29], creates a relatively larger change in capacitance. Hence it is easier to sense but its response is nonlinear. In this case, the capacitance change is inversely proportional to the square of the finger gap, thus causing a large capacitance change with acceleration. The nonlinearity can be reduced if the displacement is made very small compared with the gap spacing. Linear output also simplifies the implementation of the readout circuit. Figure 3b shows the implementation of a gap change accelerometer. The configuration is differential. Figure 3c shows the equivalent circuit diagram, which is common for gap and area changeable accelerometers.

**Figure 4:**
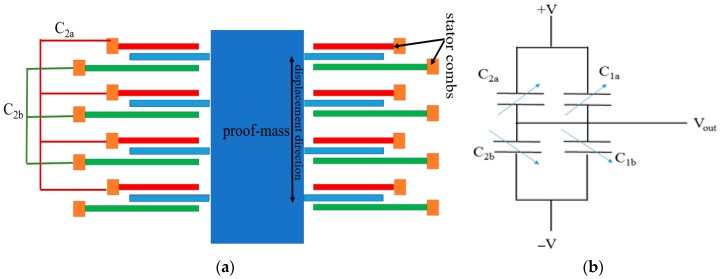
Sensing scheme of (**a**) gap changeable fully differential accelerometer (**b**) equivalent circuit.

There is also room for further improvement in gap-change accelerometers using interdigitated fingers [30,31]. Figure 4a illustrates the block diagram of a gap-change accelerometer using inter-digitated fingers. The fabrication of interdigitated fingers is complex because it requires isolation between the top and bottom stator electrodes and wiring resources to connect electrode contacts. All the top stator fingers are wired together (red fingers) to form a single capacitance plate. Similarly, all the bottom fingers are wired together (green fingers) to form another single capacitance plate. This is done so as to form a fully differential capacitive bridge (Figure 4b) in order to improve sensitivity and for canceling the offsets. 

**Figure 5:**
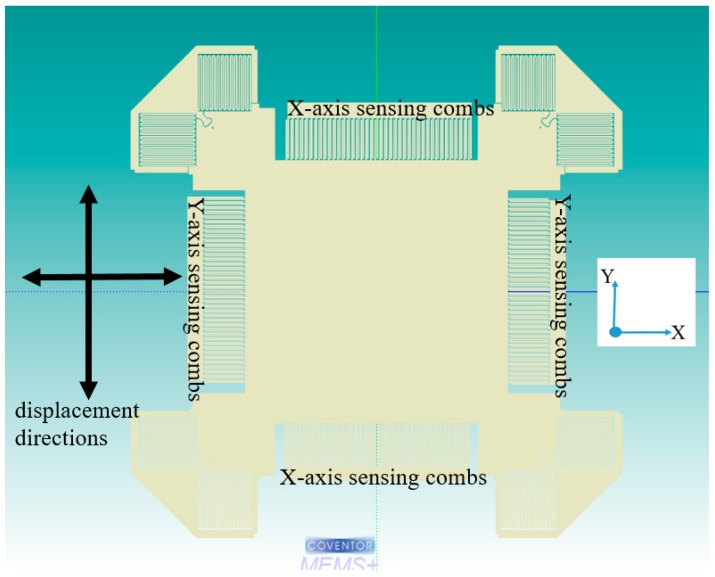
Dual axis gap change differential accelerometer.

The working of two-axis, in-plane accelerometer with a single proof mass is similar to one-axis accelerometer. However, the suspension system is designed to facilitate the displacement in the X- as well as Y-direction while having dedicated combs to sense both in-plane accelerations. Due to this, there is high cross-axis sensitivity along the X- and Y-axis. However, new methodologies are currently being developed to overcome this challenge [32]. As with the one-axis accelerometers, the 2-axis accelerometers can be area-change, gap-change, partially differential or fully differential. Figure 5 shows a sample of 2-axis accelerometer [33]. It is a gap change differential accelerometer. The Y-axis acceleration creates a gap change and therefore a capacitance changes in the Y-axis combs while the X-axis acceleration creates capacitance change in the X-axis combs. Voltages in the X- and Y-axis comb electrodes are modulated with different frequencies and demodulated at the output to measure the in-plane acceleration. 

#### 5.1.2. Z-Axis Capacitive Accelerometers

**Figure 6:**
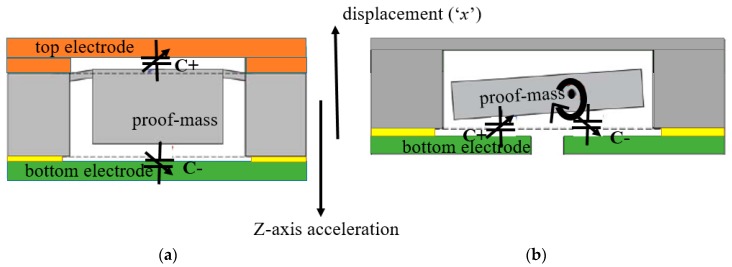
Sensing scheme of (**a**) vertical Z-axis accelerometer (**b**) torsional Z-axis accelerometer.

**Figure 7:**
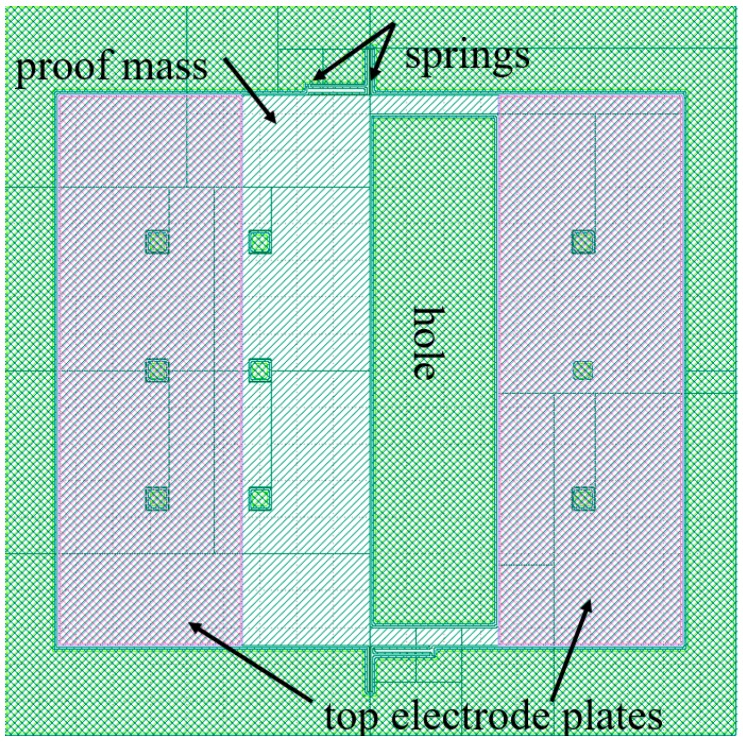
Layout of a torsional Z-axis accelerometer [31].

For sensing out-of-plane acceleration, the arrangement of a large single-stator electrode separated from the proof mass by narrow air gaps is generally used. Here, the entire proof mass acts as a rotor electrode. This stator and rotor pair acts as a parallel plate capacitor creating a capacitance change with out-of-plane displacement under applied acceleration. However, this arrangement is non-differential. In order to create a differential capacitance, two electrode plates are used [34,35,36]. Figure 6a shows such an arrangement with the proof-mass suspended between the top and bottom stator plates. When acceleration is applied, one stator gap decreases, thus increasing capacitance C+ while the other stator gap increases, thus decreasing capacitance C−. It is also possible to create differential capacitance changes with a single electrode plate. Figure 6b demonstrates a torsional Z-axis accelerometer with a single electrode plate. Here torsional springs are used to displace a non-uniform proof mass, that is, a proof mass with a nonuniform mass distribution creating a heavy side and a light side. Such non-uniform mass distribution is typically achieved by a non-uniform perforation of the proof mass using etching techniques (Figure 7). The Z-axis acceleration creates a torsional see-saw motion that results in a differential capacitance change [37,38].

## 6. Development of Monolithic Multi-Axis Capacitive Accelerometers

### 6.1. Multiple Proof-Mass Monolithic Integrated Accelerometers

Table 1 summarizes the performance of tri-axial multiple proof-mass accelerometers.

One of the simplest methods to design a three-axis accelerometer is to fabricate three individual accelerometers monolithically on a single chip. One of the earlier designs using this method was reported by M. Lemkin and B.E. Boser in 1999 [8]. The device consists of three individual proof masses, each measuring acceleration in a particular direction, fabricated using surface micromachining. The configuration used is differential (half-bridge). The X-direction (and Y-direction) sensing proof-mass uses comb fingers while for Z-axis sensing, a reference structure is attached to the substrate. The overall die size is 4 mm × 4 mm, including the readout electronics. The three individual proof masses are designed to be very small which resulted in high Brownian noise. One main advantage of Lemkin-and-Boser design is the use of a sigma-delta (ΣΔ) Modulated force-feedback loop to provide the output in digital form. In order to stabilize the proof mass after acceleration, a control signal in a negative feedback loop is used. Thus, through the control and stabilization of deflections, measurement nonlinearities are minimized. This is because feedback control extends the bandwidth of the sensor beyond its natural frequency. However, this design is not suitable for high ‘*g*’ applications. Since in high ‘*g*’, the force generated is not sufficient to bring the proof mass back into equilibrium.

In the same year, Y. Matsumoto and his collaborators demonstrated an accelerometer using an SOI fabrication process [39]. The double challenge they addressed is that of after-rinse stiction during the fabrication process and in-use stiction during operation whether it is due to high ‘g’ shock accelerations or high bias electrostatic forces due to applied voltages at the stators. Stiction is caused when rotor plates come in contact with the stator plates, resulting in output saturation and possibly permanent failure. In [39], the authors added a photoresist-buried plug and a side stopper, which removes ‘after-rinse stiction’ resulting in a more than 90% manufacturing yield. A fluorocarbon film with plasma polymerization has been used to prevent in-use stiction [40].

S. Butefisch et al. reported a three-axis bulk micromachined accelerometer (four prototypes) with four proof masses oriented orthogonal to each other [41]. Among these designs, one has the proof mass suspended by a single beam while in other a modified proof mass (triangular shape) is suspended with stiffer suspension (double beam). The latter design is of higher quality due to low cross-axis effects. The remaining two designs are improvements of the single-beam and double-beam designs. Three proof masses, each rotated by 90° in the wafer plane, were sufficient to detect acceleration in all directions, making the forth one redundant. Most probably the fourth proof mass is used to make the design more symmetric. Each proof mass measures (length × width × height) 1000 μm × 1000 μm × 300 μm, making the overall die size without the readout circuits 9000 μm × 9000 μm × 1300 μm. Some compensation schemes are used to reduce the size but still the device footprint is quite large. As the size of an individual proof mass is large, the device sensitivity is high at about 210 mV/g and 990 mV/g for a single beam and a double beam, respectively. Other exemplary designs using four proof masses are reported in [42,43] where a more compact accelerometer (2.5 mm × 2.0 mm × 6 μm) with highly symmetric sensitivity is proposed. This was at the expense of a maximum sensitivity of 1.51 fF/g, which is very low. 

A low-noise three-axis accelerometer integrating three individual proof masses was reported in [44]. The fabrication was based on both bulk and surface micromachining. By using this process, the authors of [44] were able to fabricate a thick device (475 μm) with narrow sense gap (<1.5 μm). Due to the thick structural layer and large device size, the total measured noise floor was 1.6 μg/$\sqrt{\mathrm{Hz}}$ for the X- and Y-direction and 1.08 μg/$\sqrt{\mathrm{Hz}}$ for Z-direction. The work of [44] is an integration of the authors’ previously reported in-plane accelerometer [45] and out-of-plane accelerometer [46].

In 2013, Y.C. Liu et al. have demonstrated monolithic three-axis accelerometer with multiplexed read-out circuit [47]. Due to the tight integration, the authors were able to achieve a smaller chip size. Prior to their three-axis accelerometer chip, the three-axis readout circuitry consisted of three different circuits, each connected to a proof-mass for one-axis sensing. This triplication of readout circuits results in an increase of not only the overall footprint but also of the power consumption. The in-plane acceleration is detected by comb fingers and the out-of-plane acceleration is detected using top and bottom electrode plates. The wiring is done to implement fully differential configuration which enhances the signal-to-noise ratio (SNR).

A sandwich three-axis bulk-micromachined accelerometer with three individual proof masses is proposed by S. Tez and T. Akin [48,49]. The design consists of comb fingers proof masses for in-plane sensing and electrode plates (top and bottom) for Z-axis sensing. The overall die size is 12 mm × 7 mm × 1 mm and the structural thickness of the device is 35 μm. The main focus of [48,49] is to reduce the cross-axis sensitivity and achieve low noise in a reasonable measurement range. A Double-Glass, Modified Silicon-on-Glass (DGM-SOG) process is used for fabrication. Due to the multiple stacking of glass-silicon-glass, individual in-plane and out-of-plane proof–masses are implemented. The top glass layer also acts as top electrode for the Z-axis proof mass. The same authors proposed a similar sandwiched, three-axis accelerometer [50] where the Z-axis proof mass (2 mm × 2 mm) and its electrode area are perforated to reduce damping. Again, three individual proof masses are used to sense acceleration in three directions. The lateral accelerometer has combs attached to its proof mass for in-plane sensing. Two such proof-masses (2.7 mm × 4.2 mm) are used that are oriented orthogonally to each other.

### 6.2. Single-Proof-Mass 3-Axial Accelerometers

Table 2 summarizes the design characteristics of all the single proof-mass accelerometers surveyed in this section.

One of the earliest demonstrations of using a single proof mass for three-axis sensing was performed by T. Mineta et al. [51]. The structure utilizes a bulk-micromachined, Glass-Silicon-Glass process with a sensor made of a Pyrex glass plate. The sensor has no comb fingers and only electrode plates are used for sensing both in-plane and out-of-plane acceleration. The X and Y direction acceleration cause tilting in the proof mass and the capacitance is changed with respect to the fixed electrode plates. In turn, the Z-axis acceleration causes a parallel shift. The overall chip size was 10 mm × 10 mm × 1 mm with the best achieved sensitivity being 40 mV/g. This is likely due to due to the low performance of the readout circuit. Furthermore, the cross-axis sensitivity is as high as 10%.

A surface-micromachined, single, proof-mass three-axis accelerometer with integrated electronics is reported by Lemkin and Boser [10]. The thickness of the device is 2.3 μm. It uses comb fingers for in-plane acceleration detection and one bottom electrode plate for Z-axis acceleration sensing. Comb fingers are laid in the common centroid geometry, which causes off-axis acceleration to be rejected as a common-mode, first-order signal. Furthermore, quad symmetry of the proof mass around the Z-axis is adopted to minimize cross-axis sensitivity. The design has equal compliance in the three directions with almost equal resonant frequencies. The proof mass size is 500 μm × 500 μm. The chip consists of three separate readout circuits for X-, Y- and Z-axis, yielding an overall size of 4 mm × 4 mm. In order to have high performance, the operation is closed loop, i.e., there are three individuals ΣΔ feedback loops with three readout circuits designed for the three proof masses. The results indicate a sensitivity of 0.24 fF/g for in-plane motion and 0.82 fF/g for out-of the plane motion with the maximum noise floor being 0.76 mg/$\sqrt{\mathrm{Hz}}$. The maximum cross-axis sensitivity is only 1.58% (as inferred from [10]). Due to the use of very small proof masses the device suffers from poor sensitivity and high noise.

A theoretically zero-cross-axis sensitive, single proof-mass, bulk-micromachined three-axis accelerometer is reported by Li et al. [52]. This is accomplished by using a highly symmetrical quad beam structure. There are no comb fingers in the design, and all the three-axis sensing is implemented by placing electrode plates on the top of the proof mass. The acceleration in the Z-direction causes the proof mass to move in the Z-axis while X-directional acceleration causes a rotation around the Y-axis and translation along the X-axis. The capacitances are changed with respect to fixed electrode plates placed on the top. The proof mass measures 1.8 mm × 1.8 mm in size with a structural thickness of 0.4 mm. The theoretical sensitivity is around 6–8 fF/g, which corresponds to 30–37 mV/g of measurements. The maximum cross-axis sensitivity was found to be less than 5%.

In 2003, H. Xie et al. have proposed a very compact, monolithically integrated three-axis accelerometer with a readout circuit [53]. The design consists of a large outer proof-mass in which the Z-axis proof -mass is embedded. The comb fingers are placed all around the proof mass for in-plane sensing but inside the proof mass for Z-axis sensing. The sensor uses the side-wall capacitance of the comb fingers to detect three-axis acceleration. In order to create a side-wall capacitance, three metal lines are used and are interconnected to form fully differential bridge configuration. The design uses a single crystalline silicon (SCS) CMOS-MEMS process for to achieve high resolution with a small size. The overall die measures only 1 mm × 1 mm and is able to calculate a very low-noise floor of only 50 μg/$\sqrt{\mathrm{Hz}}$. However, the design uses Al/SiO_2_ thin film spring beams for suspending the Z-axis proof-mass, thus making it more sensitive to temperature. Furthermore, the etching steps to create electrical isolation introduce undercuts on the sensing combs. In a MEMS accelerometer, the undercut problem is quite common and may be due to a variety of reasons [54]. This causes the capacitive gaps to increase between the rotor and stator combs.

H. Qu et al. have addressed the undercut and thermal sensitivity problems and reported their findings in [55]. They have used the same SCS-based CMOS-MEMS process as above. In order to avoid undercuts they have sacrificed one interconnect layer. The design consists of crab-leg suspended outer proof-mass for lateral sensing with an unbalanced proof-mass embedded inside it and suspended using a torsional spring. Again, capacitive combs wired to form a side-wall capacitance are used in a fully differential configuration. The design has nonetheless suffered undesirable undercuts due to overheating. The same authors have further addressed these issues and reported on their results in [56] where they have demonstrated a much more robust three-axis accelerometer with a readout circuit. In particular, they have used the same SCS CMOS-MEMS process and mitigated the above problems by improving the DRIE post-processing. The design was also identical with some changes in the parameters (spring length, proof-mass area…).

In 2009, Analog Devices reported a very low-cost three-axis accelerometer for consumer electronics [57]. The proposed method to reduce the cost was to use a single proof-mass accelerometer for the three-axis sensing. Also, a two-chip solution was chosen in which instead of monolithically integrating the accelerometer with readout circuit, separate chips for MEMS and electronics were used. The Analog Devices accelerometer was known as ADXL335. For fabricating this MEMS sensor, a new process was developed based on surface micromachining. From comprehending the chip micrograph, it can be concluded that comb fingers are used for in-plane sensing. For Z-axis sensing, the proof mass acts as one electrode plate, which changes the capacitance with respect to a reference electrode plate, making Z-axis sensing not fully differential. In an overall chip size of 4 mm × 4 mm × 1.45 mm, the device achieved a scale factor of 300 mV/g with a noise-limited resolution of 150 μg/$\sqrt{\mathrm{Hz}}$.

An implementation of a novel single proof-mass three-axis accelerometer was reported by C.M. Sun et al. [58]. In their approach, one proof mass (Z-axis) and two supporting frames (X- and Y-axis) are used. There are an inner proof mass for Z-axis sensing, intermediate frame for Y-axis sensing and outer frame for X-axis sensing. The inner proof mass is connected to the Y-axis frame using V-shaped springs. The Y-axis frame is connected to the X-axis frame using serpentine springs, and the X-axis frame is connected to the substrate using the same type of serpentine springs. These two sets of springs are flexible only in one direction to reduce cross-axis sensitivity. Similarly, the V-shaped Z-axis springs contribute to the reduction of cross-axis sensitivity. There are three sets of comb fingers that are micromachined on the X-, Y-, and Z-proof masses. The intermediate proof mass acts as an outer frame for the inner proof mass, and the X-axis proof mass acts as an outer frame for intermediate proof mass. The entire sensing is through gap-change comb fingers with no electrode plates being used. The Z-proof mass is designed to move in an out-of-plane direction with Z-axis acceleration causing a capacitance change in the comb fingers. Theoretically, any other acceleration (X- or Y-axis) causes no capacitance change in the Z-electrode combs and therefore no cross-axis sensitivity. The same applies for the in-plane motion of the inner and outer frames. The overall chip size along with the readout circuit is 1.78 × 1.38 mm^2^. In the acceleration range of 0.8–6 g, the results indicate a sensitivity of 0.53 mV/g, 0.28 mV/g and 0.2 mV/g for the X-, Y- and Z-axis respectively. The cross-axis sensitivity ranges from 1–8.3% and the nonlinearity is between 2.5% and 3.5% for all the three axes. The Z-axis proof mass is the smallest causing a high noise floor of 357 mg/$\sqrt{\mathrm{Hz}},$ followed by the Y-axis (271 mg/$\sqrt{\mathrm{Hz}}$) and the X-axis (120 mg/$\sqrt{\mathrm{Hz}}$).

A compact three-axis accelerometer with very low cross-axis sensitivity was reported by Y.W. Hsu et al. in [59]. Three spring-mass systems were integrated into one structure using linkage springs with an overall foot-print of 1.3 × 1.28 mm^2^. Silicon-On-Glass (SOG) bulk micromachining was used to fabricate the sensor. An inner proof mass is used for the Y-direction, an intermediate proof mass for the X-direction, and outer proof mass for the Z-direction. The in-plane sensing is done using comb fingers while for the out-of-plane Z-axis sensing, two electrode plates are used. With the Z-axis acceleration, the out-of-balance proof mass undergoes a torsional movement that generates a capacitive difference with respect to reference plates. The device is symmetric and has a sensitivity of 1.4442 V/g, 1.241 V/g, and 1.434 V/g in X-, Y-. and Z-direction, respectively. The noise floor and cross-axis sensitivity for the in-plane X- and Y-direction are 138 μg/$\sqrt{\mathrm{Hz}}$ (0.28%) and 159 μg/$\sqrt{\mathrm{Hz}}$ (0.7%), respectively, while for the Z-direction, the noise floor is 49 μg/$\sqrt{\mathrm{Hz}}$ (0.54%). The accelerometer is packaged with readout circuits and measures 4 mm × 4 mm × 1.2 mm. This design has achieved excellent performance figures that are attributed to the DRIE process with high aspect ratio and a highly symmetric design.

A very compact three-axis accelerometer (400 μm × 400 μm) was reported by M.H. Tsai et al. [60]. In this design, gap-change comb fingers are used to sense acceleration is each of the three directions. The comb fingers are distributed not only along the length and width of the proof mass but also along its thickness. This arrangement offers a larger number of fingers in a small space with the vertical fingers drastically improving sensitivity in the Z-direction. Further, the process is rich in interconnect resources that are used to connect the fingers in an interdigitated fashion in order to create fully differential configurations for all the three directions. The sensitivity is close to 15 mV/g, which corresponds to a capacitance change of approximately 2.6 fF/g. The noise floor is 2.1 mg/$\sqrt{\mathrm{Hz}}$ and the maximum cross-axis sensitivity is less than 6.6%.

A novel three-axis polysilicon rib proof-mass accelerometer was proposed by S-C Lo et al. [61]. The design is implemented using two poly-Si trench refill processes, which provides comb electrodes with high aspect ratio, thus increasing the sensing capacitance by 30 folds. For sensing in-plane acceleration, gap-change comb fingers are used, while for out-of-plane sensing, gap-change plate electrodes are used. The sensing is differential in all three directions. For Z-axis sensing, a novel method is implemented which uses movable and fixed lower and upper electrodes.

A three-axis accelerometer specifically designed for an inertial measurement unit (IMU) was reported by D.E. Serrano et al. [62]. In their design, a three-axis pendulum accelerometer is proposed to operate in vacuum. The rationale of this proposal is based on the fact that the IMU gyroscope must be packaged under vacuum, and so the integrated accelerometer itself can be packaged under the same condition. The sensor is designed for the quasi-static domain which requires high damping. This is achieved by increasing the squeeze-film damping through the reduction of the comb finger gaps. The design consists of a pendulum-like structure composed of a 450 μm × 450 μm × 40 μm single-crystal silicon proof mass anchored to the substrate by a cross-shaped polysilicon spring. This type of structure is said to be effective in reducing the footprint. The tethers that compose the spring are attached to the mass using a self-aligned process that prevents offsets in the center-of-mass. Such offsets are strong contributors to cross-axis sensitivity. Four pick-off electrodes placed on the top of the moving structure are multiplexed to read out changes in capacitance generated by the X-, Y- and Z-axis components. In the presence of acceleration along the X-axis, the tethers act as torsional springs, allowing the mass to tilt. This causes a differential change in capacitance.

### 6.3. Comparison of Single-Proof-Mass and Multiple-Proof-Mass Accelerometers

The performance of accelerometers generally scales with device size. The larger devices have higher sensitivity and lower noise floor. Therefore, in order to effectively compare accelerometers, we use normalized metrics such as sensitivity per unit area and noise-area product. For top performing sensors, the value of sensitivity per unit area should be high while the value of the noise-area product should be low. Some accelerometers have different sensitivities and noise figures along different axes. Since we are comparing three-axis accelerometers we will consider the lowest sensitivity and highest noise floor reported. The sensitivity is either expressed in fF/g or mV/g. For most of the devices, the datasheet sensitivity is given in mV/g. The mV/g gain of the off-the-shelf readout circuit MS3110 IC is used to convert the capacitive sensitivity (fF/g) into output voltage sensitivity (mV/g) for the accelerometers where voltage sensitivity (mV/g) is not reported. The output voltage of MS3110 IC is given by:

$$V_{out}=Gain*V2PS*1.14*\frac{\Delta C}{C_{F}}+V_{ref}$$

where

*Gain* = 2 or 4 (we will take 2 for our calculation)
*V2PS* = 2.25
ΔC = Capacitive Sensitivity in fF/g
$C_{F}$ = 1.5 pF
$V_{ref}$ = 0.5 or 2.25 (this is an offset that we will ignore in our comparisons)

Table 3 gives a comparison overview of various single proof-mass and multiple-proof-mass accelerometers. For a single-axis accelerometer, Chae et al. [44] has the highest sensitivity per unit area and lowest noise-area product. This was made possible by fabricating devices using a process that uses both surface and bulk micromachining techniques. The next best performance is demonstrated by the device reported in [47]. It has used a multiplexed readout circuit for reducing the overall footprint.

In the case of single proof-mass accelerometers, Tsai et al. [60] has maximum sensitivity per unit area while [62] has a minimum noise area product. The accelerometer of [61] uses a novel method to fabricate comb fingers in the thickness direction, which improves Z-axis sensitivity. The vacuum packaging in the case of [62] has helped the device to achieve a minimum noise-area product. The main problem which is faced by most single-proof mass accelerometers is with respect to Z-axis sensing. The overall performance is slightly lower than expected due to low sensitivity and high noise for Z-direction sensing.

## 7. Conclusions

In this paper, we have given an overview of monolithic, multi-axis accelerometers. We have discussed various challenges associated with multi-axis sensor design and fabrication and have provided an overview of accelerometer principles, with focus on the design options of the proof mass, sensing comb elements, fabrication process, and read out circuitry. Research on monolithic three-axis accelerometer has been on-going since 1996, and one can observe significant progress has been achieved. From MEMS accelerometers with large footprint, nonlinear, high-noise, and high cross-axis sensitivity devices, the technology has evolved into devices that are compact, highly linear, with high sensitivity, low cross-axis sensitivity, and low μg resolution.

Our literature survey has shown that the majority of accelerometers which use a single proof mass for sensing three-axis acceleration are of very small footprint and are low cost. Unfortunately, with small size come undesirable effects such as undercut of comb fingers during electrical routing. However, such effects along with nonlinearity, high cross-axis sensitivity, and noise may be solved with various innovative techniques already proven to be effective. Maintaining device symmetry was one of the targets for most of the reported devices to reduce off-axis sensitivity. In addition, to this, the concept of embedding the Z-axis proof mass in an XY-sensing frame was found to be widely used. This type of interconnected structures is structurally simpler in terms of suspension design and reduces the overall complexity. However, the use of small Z-axis proof mass increases the Brownian noise in the out-of-plane direction, thus lowering overall performance metrics.

On the other end of the design spectrum, the majority of multiple proof-mass, monolithic accelerometers with large device footprints have superior sensitivity, linearity, and noise floor. The main problem with multiple proof-mass designs is the large size required to obtain good performance. Conversely, most devices with small footprint suffer from poor sensitivity and high noise floor. 

## Figures and Tables

**Table 1:** Performance summary of tri-axial multiple proof-mass accelerometers.

Ref	Year	Author	Device Size (mm × mm)	Range (‘±g’)	Sensitivity X, Y, and Z	$\mathrm{Noise}\;(\mu g/\sqrt{Hz})\;X,\;Y,\;\mathrm{and}\;Z$	Nonlinearity X, Y, and Z	Cross-Axis Sensitivity X, Y, and Z
[8]	1999	Lemkin, M.	4 × 4 (including read out)	1.9	Digital Output (0.4 fF/bit)	110, 160, 990	-	-
[39]	1999	Matsumoto, Y.	5 × 5	-	25 fF/g, 25 fF/g, 100 fF/g			<10%
[41]	2000	Butefisch, S.	9 × 9	-	210 mV/g	-	R^2^ = 0.997	-
					990 mV/g		R^2^ = 0.99	
[43]	2005	Rodjegard, H.	2.5 × 2	-	1.27 fF/g, 1.27 fF/g, 0.82 fF/g	-	-	0.12 fF/g
[44]	2005	Chae, J.	7 × 9	1	6.8 pF/g, 6.8 pF/g, 2.9 pF/g	1.6, 1.6, 1.08	-	-
[47]	2013	Liu, Y.C.	1.57 × 1.73	0.01~2	105 mV/g, 127 mV/g, 58 mV/g	400, 210, 940	1%, 0.5%, 2.4%	3%, 2.3%, 8.8%
[49]	2015	Tez, S.	12 × 7	10 (X, Y) +12, −7 (Z)	-	5.4, 5.5, 12.6	0.34%, 0.28%, 0.41%	<1%
[50]	2016	Aydemir, A.	11.8 × 4.8	4	70.2 mV/g, 70.4 mV/g, 21.6 mV/g	13.9, 13.2, 17.8	0.26%, 0.28%, 0.3%	<1%
				71 (X, Y) 231 (Z) estimated				

**Table 2:** Performance summary of three-axis single proof-mass accelerometers.

Ref	Year	Author	Device Size (mm × mm)	Range (‘±g’)	Sensitivity X, Y, and Z	$\mathrm{Noise}\;(\mu g/\sqrt{Hz})\;X,\;Y,\;\mathrm{and}\;Z$	Nonlinearity X, Y, and Z	Cross-Axis Sensitivity X, Y, and Z
[51]	1996	Mineta, T.	10 × 10	-	-	-	-	10%
[10]	1997	Lemkin, M.A.	4 × 4 (including read out)	11-X-axis, 11-Y-axis, 5.5-Z-axis	0.24 fF/g, 0.24 fF/g, 0.82 fF/g	730, 730, 760	-	1.58% (calculated)
[52]	2001	Li, G.	1.8 × 1.8 (only proof mass)	-	30 mV/g, 30 mV/g, 37 mV/g	-	-	<5%
[53]	2003	Xie, H.	1 × 1 (including readout)	-	-	50 (estimated)	-	-
[56]	2008	Qu, H.	4 × 4 (including readout)	1	520 mV/g, 460 mV/g, 320 mV/g	12, 14, 110	-	2.38%, 2.26%, 4.73% Maximum values
[57]	2009	Hollocher, D.	4 × 4 (including read out)	3	300 mV/g	150, 150, 300	0.3%	1%
[58]	2010	Sun, C.M.	1.78 × 1.78 (including read out)	0.8~6	0.53 mV/g, 0.28 mV/g, 0.2 mV/g	120,000, 271,000, 357,000	2.64%, 3.15%, 3.36%	<7.46%, <8.05%, <8.33% Max values
[59]	2010	Hsu, Y.W.	1.3 × 1.28	1	1.44 mV/g, 1.24 mV/g, 1.4 mV/g	138, 159, 49	0.52%, 0.56%, 0.24%	0.28%, 0.7%, 0.54% Max values
[60]	2012	Tsai, M.H.	0.4 × 0.4 (only proof mass)	0~1	14.7 mV/g, 15.4 mV/g, 14.6 mV/g	2100, 2000, 2100	3.2%, 1.4%, 2.8%	6.6%, 5.4%, 5.3% Maximum values
[61]	2013	Lo, S.C.	1.7 × 1.7 (only proof mass)	0.1~3	4.31 mV/g, 4.3 mV/g, 3.48 mV/g	-	2.72%, 2.57%, 2.91%	6.8%, 6.8%, 9.0%
[62]	2014	Serrano, D.E.	0.45 × 0.45 (only proof mass)	6	6 mV/g, 5 mV/g, 11 mV/g	13, 13, 30	0.5%, 0.5%, 1%	3% (maximum)

**Table 3:** Comparison of accelerometers.

Multiple Proof-Mass Accelerometers				Single Proof-Mass Accelerometers			
Ref	Year	Sensitivity/Area (mV/mm^2^)	$\mathrm{Noise}\;\times \mathrm{Area}\;(\mu g\;{\mathrm{mm}}^{2}/\sqrt{Hz})$	Ref	Year	Sensitivity/Area (mV/mm^2^)	$\mathrm{Noise}\;\times \;\mathrm{Area}\;(\mu g\;{\mathrm{mm}}^{2}/\sqrt{Hz})$
[8]	1999	-	15,840	[10]	1997	0.0513	12,160
[39]	1999	3.42	-	[52]	2001	9.259	-
[41]	2000	2.59	-	[53]	2003	-	50
[43]	2005	0.56	-	[56]	2008	20	1760
[44]	2005	157	100	[57]	2009	18	4800
[47]	2013	21.35	2553	[58]	2010	0.06	1,131,118
[49]	2015	-	1058	[59]	2010	0.745	264
[50]	2016	0.381	1008	[60]	2012	91.25	336
-	-	-	-	[61]	2013	1.204	-
-	-	-	-	[62]	2014	24	6
